# Strategic Priorities for Implementation of Father-Inclusive Practice in Mental Health Services for Children and Families: A Delphi Expert Consensus Study

**DOI:** 10.1007/s10488-022-01222-1

**Published:** 2022-12-19

**Authors:** Marek B. Baran, Vilas Sawrikar

**Affiliations:** 1 School of Health in Social Science, University of Edinburgh, Edinburgh, UK; 2 NHS Forth Valley, Stirling, UK

**Keywords:** Delphi method, Barriers and facilitators, Father-inclusive practice, Implementation, Organizational context, Child and family services

## Abstract

The aim of this study was to investigate expert consensus on barriers and facilitators to the organizational implementation of Father-Inclusive Practice (FIP) in child and family services to establish strategic priorities for implementation. An international panel of 56 experts in child and family service provision and father inclusion were surveyed using the Delphi technique. Three online questionnaires were used to gather opinions and measure experts’ levels of agreement in regard to factors that enable or hinder the organizational implementation of FIP. Survey design, analysis and interpretation was guided by the Consolidated Framework for Implementation Research (CFIR). Consensus was achieved for 46.4% (n = 13) statements. Eight barriers and five facilitators were identified as strategic priorities to organizational implementation of FIP. The key factors were related to the following CFIR themes: leadership engagement, access to information and knowledge, implementation climate, structural characteristics, networks and communication, client needs and resources, external policies and incentives, and reflecting and evaluating. The study findings suggest that issues related to central prioritization, top-down organizational processes and external policy context should represent priority areas for implementation. Our results prioritise methods for improving FIP by highlighting the key areas of organizational practice to be addressed by tailored implementation strategies.

**Supplementary Information:**

The online version contains supplementary material available at 10.1007/s10488-022-01222-1.

Research indicates that fathers are underrepresented in services that provide psychological interventions aimed at improving outcomes for children and families (Dadds et al., 2018; Panter-Brick et al., 2014). Low levels of father engagement are observed in a range of contexts, including child welfare services (Gordon et al., 2012; Maxwell et al., 2012), paediatric psychology (Phares et al., 2005), and targeted treatments for childhood mental health and developmental disorders (Bögels & Phares, 2008; Fabiano, 2007; Flippin & Crais, 2011; Meadan et al., 2013). The extant research emphasises the importance of organizational efforts in improving rates of father engagement in child and family services, with recommendations promoting father-inclusive practice (FIP) in service design and delivery (see Lechowicz et al., 2019 for a review). However, low levels of implementation of FIP recommendations by organisations around the world have significantly restricted improvement in father participation in treatment (Cullen et al., 2011; Fletcher et al., 2014). To address this, the current study examined expert consensus on barriers and facilitators to the organizational implementation of FIP in child and family services using Delphi methodology. For the purpose of the current study, the term *child and family service(s)* encompasses organizations that provide programmes and interventions aimed at supporting the mental health and well-being of children and families.

Research evidence is consistent in showing that father involvement and active engagement with their child can significantly benefit child development, including cognitive development, social competence, academic achievement, levels of happiness and psychological well-being (Feldman et al., 2013; Flouri & Buchanan, 2003; Majdandžić et al., 2014; McBride et al., 2005; Pears et al., 2013; Pougnet et al., 2011). The increasing awareness of fathers’ potential to influence child well-being has led to greater recognition of the need to engage fathers in child and family mental health services for improving treatment outcomes (e.g., Maxwell et al., 2012). Indeed, greater, and longer-lasting improvements in child disruptive behaviour and emotional outcomes have been observed in interventions attended by fathers and mothers compared to those that included mothers only (Bagner & Eyberg, 2007; Lundahl et al., 2008). However, evidence from studies where parents’ attendance is reported suggests that fathers are engaged in family-based interventions much less frequently than mothers (Duhig et al., 2002; Fabiano, 2007; Lazar et al., 1991). This suggests that treatment effectiveness may be putatively diminished because of poor father engagement.

Difficulties in engaging fathers in child and family services are reported to be linked to multiple barriers. Personal and practical factors such as work commitments, availability of childcare, fathers’ own reluctance and beliefs about help-seeking, or *maternal gatekeeping* have been highlighted as potential obstacles (Salinas et al., 2011; Tully et al., 2017). Moreover, father engagement appears further complicated by cultural and societal attitudes towards fatherhood predicated on a deficit model that promotes the view of men as inadequate or less competent in their parental role (Hawkins & Dollahite, 1997). Importantly, evidence suggests that some of the most significant barriers to father engagement in family-based interventions may be related to practitioner-level and service-level issues affecting service provision (Panter-Brick et al., 2014; Tully et al., 2018). Lack of experience working with men, or ambivalent or negative staff attitudes towards fathers that prevent practitioners from effective encouragement of father involvement, are examples of practitioner-level barriers that hinder father engagement (Centre for Urban & Community Research, 2004; Russell et al., 1999). Service-level barriers relate to the lack of organizational commitment to father-inclusion, often exemplified, but not limited to, mother-oriented approaches and resources, absence of policies related to father-inclusion or biases in policy orientation, lack of adequate guidance for staff, or poor training availability (Bayley et al., 2009; Cullen et al., 2011; Fletcher et al., 2014). Taken together, research suggests that changes to organizational structure and practice can improve fathers’ engagement within services that provide mental health support for children and families (Panter-Brick et al., 2014).

Father-Inclusive Practice (FIP) is the term that has been used to describe the organizational commitment to “support men in their role as fathers, actively encourage their participation in programs, and to ensure that fathers are appropriately and equally considered in all aspects of service delivery” (Commonwealth of Australia, 2009, p. 9). FIP represents a significant step towards addressing the barriers to effective father engagement, with guidelines of implementation at both the practitioner- and organizational level to make child and family services more attractive, relevant, and accessible to fathers as prospective attendees (Panter-Brick et al., 2014; Tully et al., 2018). A comprehensive summary of key father-inclusive practice recommendations has been presented by Lechowicz et al. (2019) in a recent narrative review, where FIP-related recommendations were categorised into six broad themes: effective engagement of both parents, tackling the institutional bias and the ‘deficit-model’ of fathering, increasing the awareness of interventions among fathers, ensuring father-inclusive program content and delivery, increasing organizational support for father-inclusive practice, and provision of father-engagement training.

Despite these conceptual developments in FIP, little research exists evaluating the implementation of strategies targeting the structural, organizational barriers to father-involvement (Glynn & Dale, 2015; Tully, et al., 2018). This is particularly problematic because organizational factors are known to have a key role in determining how practitioners encourage or discourage father involvement (Cullen et al., 2011). Lechowicz et al. (2019) suggest that top-down, service-level strategies that emphasise father inclusion may enable practitioners to engage fathers more effectively, whereas lack of that support from the organisation constitutes a major barrier to father involvement (Glynn & Dale, 2015). It could therefore be argued that in absence of appropriate implementation of FIP by organisations, practitioners may find it difficult enacting father-inclusive practices.

Greater understanding of barriers and facilitators to implementing organizational support for FIP is needed. In related lines of implementation science research, studies have adopted specific frameworks for investigating barriers and facilitators of implementation to understand the context and processes that underpin successful adoption of research into real-world settings (e.g., Hanssen et al., 2021; Smith et al., 2019; Taba et al., 2012). One such framework applied in healthcare research is the Consolidated Framework for Implementation Research (CFIR; Damschroder et al., 2009), which provides standardized contextual determinants of implementation. The CFIR has been extensively applied to evaluate factors that influence the implementation process, highlighting its utility in conducting in-depth exploration of key variables that may either facilitate or hinder adoption of evidence-based guidelines within healthcare (Kirk et al., 2016). Notably, few studies using the CFIR framework have adopted Delphi methodology to assess factors influencing implementation (e.g., Havers et al., 2019; Strike et al., 2019). The usefulness of Delphi method in this context lies in ranking the importance of the various determinants of implementation based on consensus of experienced groups of experts. This helps establish a list of strategic priorities for implementing evidence-based guidelines into practice (e.g., Hackett et al., 2006; Havers et al., 2019; Mahoney et al., 2017; Sharpe et al., 2020; Yap et al., 2014).

The aim of the current study was to establish expert consensus on the barriers and facilitators that are most relevant to organizational implementation of FIP in child and family services, using Delphi methodology. The consensus was formed based on the opinions of a panel of experts in research and practice of family-focused interventions or promotion of father engagement. The use of CFIR allowed the grouping of the barriers and facilitators highlighted by experts into broader categories provided by the framework, to provide a standardized list of implementation determinants of FIP. This offered benefits in terms of greater scientific rigour and a more systematic approach to the analysis and interpretation of the results. Establishing key determinants that influence the organizational support for FIP was expected to help narrow down a list of strategic priorities that aid the translation FIP into practice. As the study utilized the Delphi method, which is an exploratory technique that does not lend itself to hypothesis testing (Birko et al., 2015), there were no a priori predictions regarding study outcomes.

## Methods

### Design and Procedure

Guided by the Delphi method, we conducted three iterative survey rounds to gather and aggregate responses with the aim of establishing a collective agreement among a group of selected experts (Hasson et al., 2000; Jones & Hunter, 1995). Three iterations are considered sufficient to reach consensus, enabling adequate reflection on group responses, and helping to attain stability in responses (Iqbal & Pipon-Young, 2009; Linstone & Turoff, 1975). Survey rounds were conducted between April 2021 and September 2021. Identified experts were directly invited to take part in the study via email. An information sheet was sent with the email, providing an outline of the aims of the research, selection criteria, the extent and timing of expected involvement, as well as the voluntary nature of participation. Identified experts were requested to click on a link to the Statement of Consent if they were interested in taking part in the study prior to starting the first survey. Experts were then directed to an online survey where they were required to complete a questionnaire battery assessing sociodemographic questions, followed by Round 1 survey questions. Demographic information included questions regarding experts’ age, gender, country of residence, occupation, current role, type of involvement and years of experience in work related to father engagement or child and family service provision. Details of the Delphi survey rounds can be found in the “Description of Delphi survey rounds and measures” section below. Responses to the online survey were collected using the Qualtrics online survey platform. Data for this study were collected following the ethical guidelines provided by the University of Edinburgh Human Research Ethics Committee.

### Participant Recruitment

Recruitment for the study consisted of purposive and snowball sampling (Hasson et al., 2000; Skulmoski et al., 2007). Delphi method relies on recruitment of *a panel of informed individuals*, commonly referred to as *experts* (McKenna, 1994). For the purpose of the current study, subjects were considered experts by reason of their knowledge and experience with the issues under investigation, capacity and willingness to participate; sufficient time to participate in the Delphi, and effective communication skills (Skulmoski et al., 2007). Overall, to be eligible for this study, experts were required to be above 18 years of age, English speakers, actively involved in formal research, practice or activism concerning the inclusion of fathers in healthcare settings, or delivery of programmes or interventions aimed at improving outcomes for children and families. Experts were identified through a range of means, from professional contacts of the research team, online searches of individuals who were associated with institutions within the area of interest or had authorship of articles relevant to the study, and by directly contacting multiple relevant institutions and professional networks. Contacted individuals were also asked to either nominate or pass study information on to other relevant professionals.

Fifty-six experts provided consent to participate in the study and answered the demographic questionnaire items. Of the 56 experts, 46 responded to Round 1 survey (82.14% response rate) and the same number (46) completed Round 2 survey (82.14%). Only the 46 individuals who completed Round 2 were invited to take part in Round 3, and of those, 44 completed the Round 3 questionnaire (95.65% response rate).

### Description of Delphi Survey Rounds and Measures

**Fig. 1:**
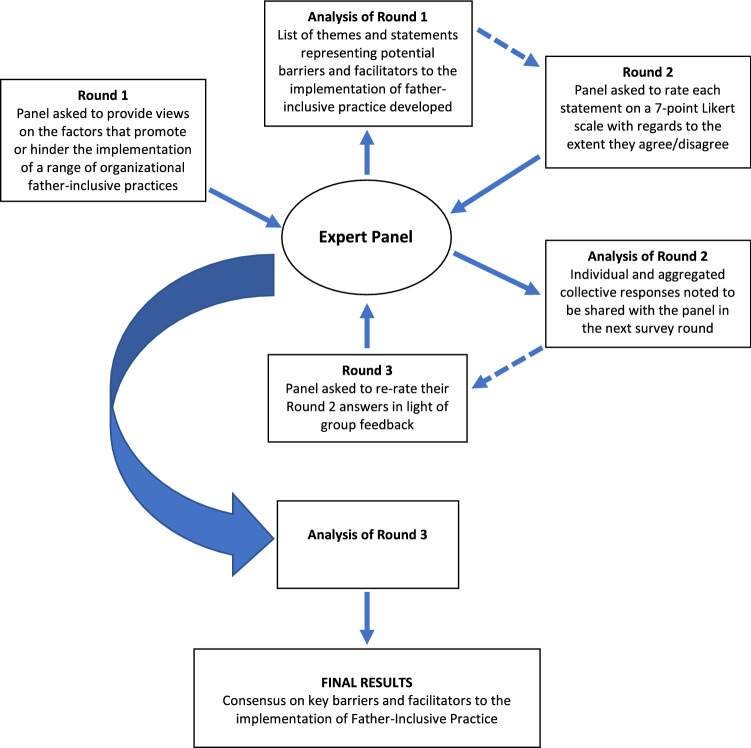
Summary of the Delphi process

A summary of the Delphi method used in the current study is presented in Fig. 1. In Round 1, experts were presented with seven open-ended questions asking for their views regarding the extent, to which selected organizational features of father-inclusive practice are implemented in child and family services, and the factors that may either hinder or support their implementation (Appendix S1, Supplementary Materials). Selected FIPs were drawn from the best practice guidelines of father engagement in family-based interventions (Panter-Brick et al., 2014, p. 1206). Participants were given a window of 3–6 weeks for the completion of the first survey. The qualitative data gathered was analysed thematically to develop specific items for questionnaires used in the subsequent survey rounds.

The second survey round (Round 2) commenced five weeks after completion of the Round 1 survey window. Participants received a questionnaire with 28 statements presenting potential barriers and facilitators to organizational implementation of father-inclusive practice (Appendix S2, Supplementary Materials). Experts were asked to indicate the extent to which they agree whether each statement constituted a barrier or a facilitator to the implementation of father-inclusive practice, on a seven-point Likert scale (‘Strongly disagree’, ‘Disagree’, ‘Somewhat disagree’, ‘Neither agree nor disagree’, ‘Somewhat agree’, ‘Agree’, ‘Strongly agree’). Seven-point Likert scale was optimal for the current study due to its high reliability, validity and discriminating power, and evidence of previous successful adoption in studies using Delphi methodology (Preston & Colman, 2000; Walsh et al., 2018). Responses to Round 2 survey were collected within a 3-week window.

Survey Round 3 was conducted two weeks after the completion of the Round 2 response collection. Round 3 represented the evaluation phase (Iqbal & Pipon-Young, 2009), whereby experts were provided with a summary of the information gathered in Round 2 and asked to reflect and reassess their initial judgments (Hsu & Sandford, 2007). Survey questions in Round 3 were the same statements as presented in Round 2. However, alongside each statement, experts were provided with a reminder of their individual reply from Round 2, and the aggregated ratings of other panel members (Appendix S3, Supplementary Materials). The experts were invited to re-evaluate their original responses in light of the group feedback and given the option to either maintain or adjust their previous rating. Experts had 3 weeks to complete survey round 3.

### Data Collection and Analysis

Anonymized data was exported from Qualtrics™ to IBM SPSS Statistics 25 for analysis at the completion of each round. Descriptive statistics were used to summarize demographic information gathered in Round 1. Analysis of the free-text responses to questions in Round 1 was subsequently supported by NVivo™. The process of converting responses from open-ended questions in Round 1 into items for Rounds 2 and 3 combined inductive and deductive thematic analytical approaches, which were carried out over two successive stages. This was done to ensure that the development of items for latter rounds captured themes relevant to father-inclusive practice. The process of thematic analysis followed Braun and Clarke’s (2006) six-phase framework, undertaken collaboratively between two researchers (MB & VS). Any differences were resolved through discussion. In the first inductive stage of analysis, phrases relating to the factors influencing implementation of father inclusive practice were coded as either barriers or facilitators, or both depending on the narrative source, and captured into descriptive themes. In the second stage of analysis, themes that had been derived in stage 1 were subsequently mapped onto the implementation determinants specified by the CFIR, and thus grouped under the five major domains that constitute the CFIR framework: intervention characteristics, outer setting, inner setting, characteristics of individuals, and process) (Fig. S1, Supplementary Materials). The five domains describe different categories of factors that may influence implementation: ‘Intervention characteristics’ refer to the features of the interventions offered by the organization; ‘Inner Setting’ encompasses the characteristics of the implementing organisation; ‘Outer Setting’ refers to external influences on implementation such as the wider context or environment; ‘Characteristics of Individuals’ relates to beliefs, knowledge and personal attributes of those involved; and ‘Process’ includes the specific implementation activities (Damschroder et al., 2009).

One theme (Remote service provision) could not be mapped on existing CFIR constructs; thus, an additional construct (Intervention Modality) was added to the CFIR domain of ‘Intervention Characteristics’. Altogether, 28 barriers and facilitators were represented in 17 CFIR constructs (Table S1, Supplementary Materials). The identified barriers and facilitators were paraphrased into the 28 individual questionnaire items used in Rounds 2 and 3.

Descriptive analysis of individual levels of agreement to items was used to establish consensus among experts from responses collected in survey round 3. Consensus was assumed to have been reached if > 70% of experts agreed (summative of agree and strongly agree) with an individual statement at the end of round 3. This criterion of consensus follows methods used in previous Delphi studies (e.g., Vernon, 2009; Veugelers et al., 2020; Vogel et al., 2019; Walsh et al., 2018). Barriers and facilitators were reported separately, with their associated consensus levels. Statements that achieved consensus of > 70% at the end of Round 3 were identified as the strategic priorities to the organizational adoption of father-inclusive practice in child and family services.

## Results

### Demographic Information

Table 1 summarises the demographic characteristics of the participants included in the analysis. The sample included experts from the United Kingdom (67.9%), United States (12.5%), Canada (12.5%), Australia (5.4%) and Belgium (1.8%). Of the 56 experts that took part, 33 (58.9%) participants identified as female and 23 (41.1%) as male. The average age of participants was 50.7 years (SD = 12.9). Those who identified primarily as research and academic professionals represented the largest group in the expert panel (28.6%, n = 16), followed by services managers and those in leadership roles (19.6%, n = 11), practitioner psychologists (19.6%, n = 11), and family support workers and coordinators (14.3%, n = 8). In terms of professional experience, a large proportion of participants reported involvement in direct clinical work (60.7%, n = 34), while 37.6% (n = 21) indicated being involved in indirect clinical service provision, particularly service leadership and managerial roles. Nearly half of all experts (44.6%, n = 25) reported engagement in academic work and 19.6% (N = 11) identified as pursuing activities related to consultancy, campaigning, and policymaking. Of all experts, 44.6% (n = 25) indicated fulfilling multiple professional roles that simultaneously covered several types of professional experience (e.g., both direct clinical and academic work). The length of time working in the relevant fields ranged from 1 to 60 years, with average years of experience of 18.57 (SD = 11.15).

**Table 1:** Characteristics of the participants included in the analysis

Demographic	Frequency	Percentage
Gender		
Female	33	58.9
Male	23	41.1
Years of relevant experience		
1–5 years	5	8.9
6–10 years	11	19.6
11–20 years	25	44.6
21–30 years	10	17.9
30+ years	5	8.9
Current role		
Academic/Researcher	16	28.6
Practitioner psychologist	11	19.6
Manager/Service lead	11	19.6
Family support worker/Coordinator	8	14.3
Nurse/Health visitor/Midwife	5	8.9
Project worker/Coordinator	3	5.4
Family therapist	1	1.8
Social worker	1	1.8
Type of involvement with the target area		
Direct client work (clinical/support)	34	60.7
Research/Academic	25	44.6
Indirect work (managerial/leadership/administrative)	21	37.5
Consultancy/Campaigning/Policymaking	11	19.6
Other: training, capacity-building	2	3.5

	Mean	Std. dev.
Age (in years)	50.7	12.9

### Consensus Analysis

Tables 2 and 3 report the statements relevant to barriers and facilitators along with their associated levels of agreement. At the end of Round 3, 13 out of the 28 (46.4%) statements achieved consensus (> 70% agreement) to be considered key strategic priorities for the organizational implementation of father-inclusive practice in child and family services. This included 8 of the 21 statements representing barriers, and 5 of the 7 statements representing facilitators. Consensus was not obtained for the remaining 18 statements.

**Table 2:** Number of responses and percentage agreement (strongly agree/agree) to Round 3 survey items relating to barriers to father-inclusive practice

CFIR domains	CFIR constructs	Barriers to father-inclusive practice	SD	D	SwD	N	SwA	A	SA	%A	Consensus achieved
Outer setting	Patient/Client needs and resources	Services having limited awareness of fathers’ needs and how to address them	0	2	1	0	7	**25**	**9**	**77.3%**	Yes
Inner setting	Access to knowledge and information	Lack of clear protocols that would help staff to adequately engage fathers	0	1	1	0	9	**23**	**10**	**75%**	Yes
Inner setting	Leadership engagement	Lack of leadership’s commitment, engagement, and support for father-inclusive practice	0	2	0	2	8	**20**	**12**	**72.7%**	Yes
Inner setting	Structural characteristics	No centralized guidance, i.e., father-inclusive practice being left at individual professional’s discretion	0	0	1	2	9	**17**	**15**	**72.7%**	Yes
Inner setting	Goals and feedback	Father inclusive practices not being linked to service targets or key performance indicators	0	0	0	3	9	**22**	**10**	**72.7%**	Yes
Inner setting	Access to knowledge and information	Lack of training and education for the workforce on how to improve father inclusion	0	0	1	2	10	**16**	**15**	**70.5%**	Yes
Inner setting	Compatibility	Father-inclusive practices being viewed as creating additional work, resulting in excessive burden for staff	0	1	2	2	8	**22**	**9**	**70.5%**	Yes
Inner setting	Relative priority	Father inclusion not being recognized as a strategic priority by services	0	0	0	0	13	**17**	**14**	**70.5%**	Yes
Characteristics of individuals	Self-efficacy	Lack of confidence among child and family professionals in engaging dads	1	1	2	3	7	14	16	68.2%	No
Inner setting	Access to knowledge and information	Lack of awareness of father-inclusive practice guidelines and recommendations	0	0	0	1	15	20	8	63.7%	No
Characteristics of individuals	Knowledge and beliefs about intervention	Staff’s assumptions and stereotypes about fathers’ role, availability or interest in child and family interventions	0	2	3	0	12	8	19	61.4%	No
Inner setting	Compatibility	Inadequate data recording systems, which don’t allow for storing and collating information that could enhance father inclusion	0	2	3	6	6	17	10	61.4%	No
Outer setting	External policy and incentives	Father-inclusive practices not being incentivized by the authorities and/or commissioners	0	0	2	4	13	14	11	56.8%	No
Inner setting	Available resources	Staff not having enough time for activities related to father-inclusive practice due to other competing demands	0	4	3	2	10	16	9	56.8%	No
Inner setting	Available resources	Lack of gender diversity in the staff teams, e.g. disproportionate percentage of female practitioners	0	1	3	5	12	5	18	52.3%	No
Inner setting	Organizational culture	General organizational culture of resistance to change and reluctance to introduce new initiatives	0	2	3	4	13	16	6	50%	No
Inner setting	Available resources	Organizations lacking sufficient financial resources to adequately fund father-inclusive practices	0	2	2	3	17	11	9	45.5%	No
Inner setting	Organizational incentives and rewards	No rewards offered from service leadership for staff to be more inclusive of fathers in their practice	1	2	0	13	10	13	5	40.9%	No
Inner setting	Available resources	Limited staff availability due to inflexible working hours	0	5	3	4	14	9	9	41%	No
Characteristics of individuals	Knowledge and beliefs about intervention	Staff’s assumptions that implementing father-inclusive practices will not lead to improved outcomes or increased father engagement	0	4	9	7	10	10	4	31.8%	No
Outer setting	External policy and incentives	Reluctance to focus attention on fathers' needs because of the drive for child and family services to be gender-neutral	0	3	4	12	16	8	1	20.5%	No

Levels of agreement for items that achieved consensus are highlighted in bold

Items presented in descending order of strength of agreement

*SD* strongly agree, *D* disagree, *SwD* somewhat disagree, *N* neither agree nor disagree, *SwA* somewhat agree, *A* agree, *SA* strongly agree, *%A* = percentage agreement

**Table 3:** Number of responses and percentage agreement (strongly agree/agree) to Round 3 survey items relating to the facilitators to father-inclusive practice

CFIR domains	CFIR constructs	Barriers to father-inclusive practice	SD	D	SwD	N	SwA	A	SA	%A	Consensus achieved
Inner setting	Networks and communication	Services actively encouraging team discussion and reflection about implementing father-inclusive practice	0	0	0	2	0	**24**	**18**	**95.4%**	Yes
Inner setting	Access to knowledge and information	Services providing opportunities for staff education and training on father-inclusive practice	0	0	0	0	2	**17**	**25**	**95.4%**	Yes
Process	Reflecting and evaluating	Services introducing clear measures to monitor their provision of father-inclusive practice	0	0	1	1	2	**23**	**17**	**90.9%**	Yes
Outer setting	External policy and incentives	Greater recognition of the importance of father-inclusive practice by the authorities and service commissioners	0	0	0	0	6	**18**	**20**	**86.4%**	Yes
Inner setting	Goals and feedback	Services introducing targets and key performance indicators related to father-inclusive practice	0	1	0	2	7	**24**	**10**	**77.2%**	Yes
Process	Champions	Services nominating champions committed to improving father inclusion	0	0	1	1	12	25	5	68.2%	No
Intervention characteristics	Intervention modality	Services providing more emphasis on remote treatment provision in efforts to better engage dads	0	0	1	8	9	23	3	59.1%	No

Levels of agreement for items that achieved consensus are highlighted in bold

Items presented in descending order of strength of agreement

*SD* strongly agree, *D* disagree, *SwD* somewhat disagree, *N* neither agree nor disagree, *SwA* somewhat agree, *A* agree, *SA* strongly agree, *%A* percentage agreement

#### Barriers to Implementing FIP

Seven out of eight barriers that reached consensus were associated with constructs belonging to the *Inner Setting* domain. Two of those barriers were related to *access to knowledge and information*. The experts agreed that lack of clear service protocols to adequately engage fathers, and lack of training and education for the workforce, were significant barriers to the implementation of FIP. Another barrier that met consensus was related to *leadership engagement*, highlighting that according to most of the experts, lack of leadership support for FIP was one of the key factors impeding its organizational adoption. Furthermore, experts were also in agreement that lack of centralized guidance, i.e., leaving the decision whether or not to engage in FIP in the hands of an individual professional, was an important structural characteristic of an organisation that hinders implementation. The other three barriers that achieved high consensus rates were related to aspects of the *Implementation Climate* construct: *Relative Priority*, *Goals and Feedback* and *Compatibility*. That is, the experts agreed that lack of strategic prioritization of FIP, absence of service targets and key performance indicators related to FIP, and the view of FIP as creating excessive burden for staff, were important barriers to FIP implementation. The only other barrier that met consensus was associated with the *Outer Setting* construct of *Patient Needs and Resources*. Experts were in agreement that the limited awareness of fathers’ needs within services is a major obstacle to the organizational implementation of FIP.

Thirteen statements did not meet expert consensus as key strategic priorities for FIP implementation. These included all three statements belonging to the *Characteristics of Individuals* domain, such as practitioners’ lack of confidence in engaging fathers, assumptions and stereotypes about fathers’ role availability or interest, or assumptions of FIP not leading to improved outcomes. Furthermore, eight statements related to the Inner Setting also did not meet consensus. For instance, none of the statements associated with *resource availability* (insufficient time due to competing demands, lack of gender diversity in staff teams, insufficient financial resources, limited staff availability) reached consensus to be considered a key barrier to FIP implementation. Other *Inner Setting* barriers that did not reach consensus threshold included: lack of awareness of FIP guidelines and recommendations, inadequate data systems, organizational resistance to change, and lack of organizational incentives and rewards. Lastly, two barriers that did not meet consensus were related to belonged to the *Outer Setting* domain. There was no agreement on the lack of incentives from authorities being a high-priority barrier to implementation of FIP. Furthermore, no consensus was reached regarding the statement that a drive towards gender-neutrality was a barrier to the organizational adoption of FIP in child and family services.

#### Facilitators to Implementing FIP

The experts reached consensus on five facilitators of FIP. Three of those belonged to the *Inner Setting* domain. Firstly, experts agreed that practices such as allowing and encouraging team discussions and reflection regarding FIP can be a major facilitator to organizational implementation of FIP. Moreover, experts recognized a need for greater access to knowledge and information about FIP among staff, as provision of education and training for staff was recognized as a key enabler of FIP. Another facilitator that met consensus was related to the services having explicit goals for introducing FIP, which could be achieved through introduction of relevant targets and key performance indicators that guide service delivery. Furthermore, one facilitator that met the consensus threshold was associated with the *External Policies and Incentives* construct of the *Outer Setting* domain. That is, experts agreed that greater recognition of the importance of FIP by the authorities and commissioners is a significant facilitator of FIP. Finally, one statement that met expert consensus as a key facilitator of FIP belonged to the *Implementation Process* domain and related to the importance of having clear measures to monitor the provision of FIP.

Only two facilitators did not reach the necessary consensus level to be considered a strategic priority for the organizational implementation of FIP. One of the statements was related to the *Implementation Process* domain and highlighted that enlisting champions for father inclusion was not viewed as a key priority for implementation of FIP in services. The second statement that did not attract high levels of agreement was related to the *Intervention Characteristics* domain. More specifically, increasing remote service provision was not highlighted as a key enabler of FIP.

## Discussion

The current study examined barriers and facilitators to organizational implementation of father inclusive practice (FIP) within services that provide interventions to improve the mental health and well-being of children and families. The purpose was to establish areas of strategic priority for future FIP implementation. Our results indicated eight barriers and five facilitators considered as implementation priorities. These were arranged across the relevant domains and constructs of the CFIR implementation framework. Most belonged to the Inner Setting domain reflecting issues of central prioritization of FIP. By contrast, the factors not identified as priority varied across multiple CFIR domains, representing practitioner-level, service-level, and external influences.

Our results suggest that one of the key overarching themes that hinders the organizational implementation of FIP is lack of central prioritisation and provision to support FIP. This result replicates and extends previous research findings that highlighted the critical importance of organizational support to greater father-inclusivity (e.g., Glynn & Dale, 2015; Tully et al., 2018). Notably, lack of leadership support, centralized guidance, and performance monitoring were identified as key barriers to implementing FIP. These findings converge with previous research, which emphasize the instrumental role of leadership in implementing new initiatives by facilitating buy-in among staff and ensuring that new processes are integrated into practice (Li et al., 2018). Conversely, research shows that leadership that is reluctant to partake in the implementation process or neglects to hold staff accountable can undermine the implementation of evidence-based practices (Lodge et al., 2017; Omer, 2012). Indeed, high staff autonomy was found to be a major barrier in our analysis, alongside previous studies, adding to evidence that without centralized guidance regarding implementing new evidence-based practices, staff may be resistant in adopting father-inclusive practice (e.g. Berta et al., 2005; Lodge et al., 2017).

Relatedly, our findings highlighted that successful implementation of FIP depends on the compatibility between FIP and the existing work processes. Experts agreed that implementing FIP is likely to be hampered in environments where it is perceived as an extra burden. Indeed, new initiatives that don’t align with the organizational norms, ways of working, and perceived needs, have less likelihood of being adopted (Greenhalgh et al., 2004). This is emphasised for initiatives viewed as complex or difficult to implement (Greenhalgh et al., 2004). While the issue of compatibility is related to lower-level operational context, it is possible that top-down influences such as effective leadership may offer ways of fostering more positive attitudes towards implementation (Aarons, 2006).

Furthermore, our study identified the lack of organizational awareness of fathers’ needs as a major obstacle to FIP implementation. This echoes the view of Rollins (2020), who recognized father awareness as the first step towards greater father-inclusivity in services. Research suggests that awareness of fathers’ needs is a factor that can be potentially addressed by adequate staff education (e.g. Humphries & Nolan, 2015; Rollins, 2020). This corresponds with our findings, which also emphasised the strategic importance of staff education and training for FIP implementation. Consequently, our results are supportive of the view that FIP training provision represents one potential strategy to remedy the lack of organizational father awareness. Moreover, our outcomes align more generally with previous research that highlights the multi-level benefits of staff training in enhancing father-inclusivity and improving organizational practices in services (e.g. Burn et al., 2019; Humphries & Nolan, 2015; Scourfield et al., 2012, 2015).

The experts in our study were explicit about top-down organizational facilitators for implementing FIP, many of them reflecting ways of addressing implementation barriers. For instance, organizational encouragement of discussion and reflection on father-inclusive practice was recognized as important for implementation. This converges with previous findings that intra-organizational communication can impact the implementation of evidence-based practice (e.g. Harvey et al., 2015; McCullough et al., 2015). More specifically, the evidence suggests that establishing systems and processes to facilitate communication about new initiatives (in this case, FIP), and utilizing various communication channels to encourage dialogue about the new initiative among staff, can contribute to implementation success (Harvey et al., 2015; Stevens et al., 2014; Vamos et al., 2017). Secondly, service targets and key performance indicators were recognized as important facilitators to FIP, suggesting that setting targets in relation to FIP has the potential to boost implementation success. In line with this, the results also highlighted that monitoring and auditing the implementation of FIP putatively encourages good practice among staff in healthcare contexts (Stevens et al., 2014; Yamada et al., 2017). Lastly, we hypothesise that the organizational willingness to implement targets and monitoring to enhance FIP may be dependent on the external policy context. Service targets and performance indicators often reflect local and national priorities that exist in the form of regulations and guidelines (Davies et al., 2021; Mendel et al., 2008). Our findings add to the evidence that systems-level support for FIP from external bodies (government and commissioners) represents an important facilitator to FIP, which may directly impact the services’ motivation to adopt new initiatives (Greenhalgh et al., 2004).

By contrast to areas identified as priority for implementation, our findings indicated that practitioner-level issues such as staff’s assumptions or confidence in engaging fathers are arguably less of a priority for the successful implementation of FIP. These findings somewhat contrast previous research emphasising practitioners in improving father engagement (e.g., Burn et al., 2019; Hecker, 1991; Vetere, 2004; Wolins, 1983). It is important to note that the lack of recognition of practitioner-level issues as a priority area is reflective of the opinion and the characteristics of the current expert sample, 40% of whom did not identify as frontline clinical staff. While some caution should be exercised when drawing inferences from this finding, the results are clear in suggesting that focusing solely on improving individual practitioner skills without putting in adequate organizational support and addressing challenges associated with external policy and social context, might not lead to improved father inclusivity. Furthermore, our results show that, contrary to findings from previous studies (e.g., Bach-Mortensen et al., 2018; Li et al., 2018), factors associated with availability of resources, such as time constraints, staffing and financial limitations, might hold relatively less strategic importance for FIP implementation. In light of our findings, we hypothesise that organizational implementation of FIP may be less resource-intensive than previously assumed. Additionally, it is possible that putting in place effective top-down strategies and processes may in fact help with adequate allocation of existing resources to facilitate implementation.

Taken together, our findings emphasise numerous interrelationships that exist between factors influencing FIP provision. We identified potential contingencies between systems-level influences, top-down organizational guidance and prioritization, leadership support, lower-level organizational processes, and practitioner competencies. Our results therefore reinforce the notion that many implementation determinants are interdependent and work synergistically to influence implementation (Li et al., 2018; Sarkies et al., 2020). Taking that into consideration, we suggest that successful organizational implementation of FIP relies on accurately identifying these interrelationships and taking them into account when coordinating future FIP implementation efforts.

### Limitations and Future Research Directions

Several limitations should be considered for future research. We recruited a very diverse sample of experts working within different systems and policy contexts to gain a wide-ranging perspective on the issue of implementation of FIP. While this increased the generalizability of our findings, one potential limitation of this approach was the possibility that barriers and facilitators may differ significantly depending on the local and national context, impacting the experts’ levels of agreement on the key factors. Therefore, one consideration for future research should be to focus on assessments of factors affecting FIP provision that target a particular service type, locality, or professional group, which could offer more context-specific insights.

Although previous research identifies snowball sampling as a valid method of identifying expert populations in Delphi studies (e.g. Skulmoski et al., 2007), we have found that applying this sampling method in the current study may have led to the inclusion of some non-experts in the sample. While the years of relevant experience reported by the sample was high (18.5), it ranged from 1 to 60, which indicates that those at the lower end may have had a more limited expertise in relation to child and family service provision. This could have influenced the robustness of our data, and therefore should be carefully considered by future research involving expert samples. This challenge could be possibly remedied by introducing more stringent minimum inclusion criteria in relation to the participants’ experience.

Due to the need to rapidly convert open-ended responses into questionnaire items, the processes of inductive coding, derivation of themes and their matching to CFIR framework in the Round 1 thematic analysis, were not done independently by the members of the research team. Although thematic analysis was not a specific aspect of our study, non-independence in coding might have introduced potential bias, thus impacting the reliability of the analysis. Allowing more time and introducing procedures to assess the inter-rater reliability would help to mitigate this risk of bias.

Although the recruited sample of experts was balanced in terms of variety of professional experience, some experts may have had a limited exposure to certain aspects of child and family service provision, such as the direct clinical work. Therefore, future research using expert samples should consider the extent to which expertise of the participants matches the focus of the study.

Pinpointing the key determinants of implementation is merely the first step towards enabling greater father-inclusivity and requires further research to identify and select appropriate implementation strategies to address the identified barriers and facilitators. This is another limitation of this approach: determinant frameworks such as the CFIR, provide insight into factors influencing implementation, but do not specify the mechanisms of change or provide support for carrying out the process of implementation. Therefore, future research should build on these findings by determining and matching discrete implementation strategies to address the CFIR-based contextual factors. This could be achieved by utilizing a range of process models designed to guide the translation of research into practice (see Nilsen, 2015 for a review), including the Expert Recommendations for Implementing Change (ERIC) compilation, which has been specifically designed to complement the CFIR framework in tailoring the implementation process (Waltz et al., 2019). Finally, due to descriptive nature of the CFIR framework, the current study offers limited insight into the possible synergistic relationships between the factors that influence the organizational implementation of FIP. Future research should address this by exploring further how the individual barriers and facilitators interact to influence the implementation of organizational practices to aid father inclusion.

## Conclusion

By establishing expert consensus on strategic priorities among the factors that enable or obstruct the organizational adoption of FIP, we provided an evidence base of the key priorities that should be considered by services aiming to enhance their father-inclusive practice. From the evidence, it was clear that more attention should be given primarily to the top-down organizational processes and practices to improve the service-level provision of FIP. The findings of this study should inform the identification and selection of appropriate implementation strategies to address the existing service-level challenges to FIP.

## Supplementary Information

Below is the link to the electronic supplementary material.

Supplementary file1 (DOCX 125 kb)

## References

[CR1] Aarons GA (2006). Transformational and transactional leadership: Association with attitudes toward evidence-based practice. Psychiatric Services.

[CR2] Bach-Mortensen AM, Lange BCL, Montgomery P (2018). Barriers and facilitators to implementing evidence-based interventions among third sector organisations: A systematic review. Implementation Science.

[CR3] Bagner DM, Eyberg SM (2007). Parent–child interaction therapy for disruptive behavior in children with mental retardation: A randomized controlled trial. Journal of Clinical Child and Adolescent Psychology.

[CR4] Bayley J, Wallace LM, Choudhry K (2009). Fathers and parenting programmes: Barriers and best practice. Community Practitioner.

[CR5] Berta W, Teare GF, Gilbart E, Ginsburg LS, Lemieux-Charles L, Davis D, Rappolt S (2005). The contingencies of organizational learning in long-term care: Factors that affect innovation adoption. Health Care Management Review.

[CR6] Birko S, Dove ES, Özdemir V (2015). A Delphi Technology Foresight Study: Mapping social construction of scientific evidence on metagenomics tests for water safety. PLoS ONE.

[CR7] Bögels S, Phares V (2008). Fathers’ role in the etiology, prevention and treatment of child anxiety: A review and new model. Clinical Psychology Review.

[CR8] Braun V, Clarke V (2006). Using thematic analysis in psychology. Qualitative Research in Psychology.

[CR9] Burn M, Tully LA, Jiang Y, Piotrowska PJ, Collins DAJ, Sargeant K, Hawes D, Moul C, Lenroot RK, Frick PJ, Anderson V, Kimonis ER, Dadds MR (2019). Evaluating practitioner training to improve competencies and organizational practices for engaging fathers in parenting interventions. Child Psychiatry & Human Development.

[CR10] Centre for Urban and Community Research (2004). Project and literature review on fatherhood for North Leyton Sure Start.

[CR11] Commonwealth of Australia (2009). Father-inclusive practice guide: A tool to support the inclusion of fathers in a holistic approach to service delivery.

[CR12] Cullen SM, Cullen MA, Band S, Davis L, Lindsay G (2011). Supporting fathers to engage with their children’s learning and education: An under-developed aspect of the Parent Support Adviser pilot. British Educational Research Journal.

[CR13] Dadds MR, Collins DAJ, Doyle FL, Tully LA, Hawes DJ, Lenroot RK, Anderson V, Frick PJ, Moul C, Kimonis ER (2018). A benchmarking study of father involvement in Australian child mental health services. PLoS ONE.

[CR14] Damschroder LJ, Aron DC, Keith RE, Kirsh SR, Alexander JA, Lowery JC (2009). Fostering implementation of health services research findings into practice: A consolidated framework for advancing implementation science. Implementation Science.

[CR15] Davies N, Atkins G, Sodhi S (2021). Using targets to improve public services.

[CR16] Duhig AM, Phares V, Birkeland RW (2002). Involvement of fathers in therapy: A survey of clinicians. Professional Psychology: Research and Practice.

[CR17] Fabiano GA (2007). Father participation in behavioral parent training for ADHD: Review and recommendations for increasing inclusion and engagement. Journal of Family Psychology.

[CR18] Feldman R, Bamberger E, Kanat-Maymon Y (2013). Parent-specific reciprocity from infancy to adolescence shapes children’s social competence and dialogical skills. Attachment & Human Development.

[CR19] Fletcher R, May C, St George J, Stoker L, Oshan M (2014). Engaging fathers: Evidence review.

[CR20] Flippin M, Crais ER (2011). The need for more effective father involvement in early autism intervention: A systematic review and recommendations. Journal of Early Intervention.

[CR21] Flouri E, Buchanan A (2003). The role of father involvement and mother involvement in adolescents’ psychological well-being. British Journal of Social Work.

[CR22] Glynn L, Dale M (2015). Engaging dads: Enhancing support for fathers through parenting programmes. Aotearoa New Zealand Social Work.

[CR23] Gordon DM, Oliveros A, Hawes SW, Iwamoto DK, Rayford BS (2012). Engaging fathers in child protection services: A review of factors and strategies across ecological systems. Children and Youth Services Review.

[CR24] Greenhalgh T, Robert G, Macfarlane F, Bate P, Kyriakidou O (2004). Diffusion of innovations in service organizations: Systematic review and recommendations. The Milbank Quarterly.

[CR25] Hackett S, Masson H, Phillips S (2006). Exploring consensus in practice with youth who are sexually abusive: Findings from a Delphi study of practitioner views in the United Kingdom and the Republic of Ireland. Child Maltreatment.

[CR26] Hanssen DJC, Ras A, Rosmalen JGM (2021). Barriers and facilitators to the implementation of interventions for medically unexplained symptoms in primary care: A modified Delphi study. Journal of Psychosomatic Research.

[CR27] Harvey G, Jas P, Walshe K (2015). Analysing organizational context: Case studies on the contribution of absorptive capacity theory to understanding inter-organizational variation in performance improvement. BMJ Quality & Safety.

[CR28] Hasson F, Keeney S, McKenna H (2000). Research guidelines for the Delphi survey technique. Journal of Advanced Nursing.

[CR29] Havers SM, Martin E, Wilson A, Hall L (2019). Implementation of government-directed policy in the hospital setting: A modified Delphi study. Health Research Policy and Systems.

[CR30] Hawkins AJ, Dollahite DC (1997). Generative fathering: Beyond deficit perspectives.

[CR31] Hecker LL (1991). Where is dad? 21 ways to involve fathers in family therapy. Journal of Family Psychotherapy.

[CR32] Hsu C-C, Sandford BA (2007). The Delphi technique: Making sense of consensus. Practical Assessment, Research & Evaluation.

[CR33] Humphries H, Nolan M (2015). Evaluation of a brief intervention to assist health visitors and community practitioners to engage with fathers as part of the healthy child initiative. Primary Health Care Research & Development.

[CR34] Iqbal S, Pipon-Young L (2009). The Delphi method. Psychologist.

[CR35] Jones J, Hunter D (1995). Consensus methods for medical and health services research. British Medical Journal.

[CR36] Kirk MA, Kelley C, Yankey N, Birken SA, Abadie B, Damschroder L (2016). A systematic review of the use of the Consolidated Framework for Implementation Research. Implementation Science.

[CR37] Lazar A, Sagi A, Fraser MW (1991). Involving fathers in social services. Children and Youth Services Review.

[CR38] Lechowicz ME, Jiang Y, Tully LA, Burn MT, Collins DAJ, Hawes DJ, Lenroot RK, Anderson V, Doyle FL, Piotrowska PJ, Frick PJ, Moul C, Kimonis ER, Dadds MR (2019). Enhancing father engagement in parenting programs: Translating research into practice recommendations. Australian Psychologist.

[CR39] Li S-A, Jeffs L, Barwick M, Stevens B (2018). Organizational contextual features that influence the implementation of evidence-based practices across healthcare settings: A systematic integrative review. Systematic Reviews.

[CR40] Linstone HA, Turoff M (1975). The Delphi method. Techniques and applications.

[CR41] Lodge AC, Kaufman L, Stevens Manser S (2017). Barriers to implementing person-centered recovery planning in public mental health organizations in Texas: Results from nine focus groups. Administration and Policy in Mental Health and Mental Health Services Research.

[CR42] Lundahl BW, Tollefson D, Risser H, Lovejoy MC (2008). A meta-analysis of father involvement in parent training. Research on Social Work Practice.

[CR43] Mahoney JE, Clemson L, Schlotthauer A, Mack KA, Shea T, Gobel V, Cech S (2017). Modified Delphi consensus to suggest key elements of *Stepping On* Falls Prevention Program. Frontiers in Public Health.

[CR44] Majdandžić M, Möller EL, de Vente W, Bögels SM, van den Boom DC (2014). Fathers’ challenging parenting behavior prevents social anxiety development in their 4-year-old children: A longitudinal observational study. Journal of Abnormal Child Psychology.

[CR45] Maxwell N, Scourfield J, Featherstone B, Holland S, Tolman R (2012). Engaging fathers in child welfare services: A narrative review of recent research evidence: Engaging fathers in child welfare services. Child & Family Social Work.

[CR46] McBride BA, Schoppe-Sullivan SJ, Ho M-H (2005). The mediating role of fathers’ school involvement on student achievement. Journal of Applied Developmental Psychology.

[CR47] McCullough MB, Chou AF, Solomon JL, Petrakis BA, Kim B, Park AM, Benedict AJ, Hamilton AB, Rose AJ (2015). The interplay of contextual elements in implementation: An ethnographic case study. BMC Health Services Research.

[CR48] McKenna HP (1994). The Delphi technique: A worthwhile research approach for nursing?. Journal of Advanced Nursing.

[CR49] Meadan H, Parette HP, Doubet S, Pattnaik J (2013). Fathers of young children with disabilities. Father involvement in young children’s lives: A global analysis.

[CR50] Mendel P, Meredith LS, Schoenbaum M, Sherbourne CD, Wells KB (2008). Interventions in organizational and community context: A framework for building evidence on dissemination and implementation in health services research. Administration and Policy in Mental Health.

[CR51] Nilsen P (2015). Making sense of implementation theories, models and frameworks. Implementation Science.

[CR52] Omer T (2012). Research utilization in a multicultural nursing setting in Saudi Arabia: Barriers and facilitators. Journal of Nursing Research.

[CR53] Panter-Brick C, Burgess A, Eggerman M, McAllister F, Pruett K, Leckman JF (2014). Practitioner review: Engaging fathers—Recommendations for a game change in parenting interventions based on a systematic review of the global evidence. Journal of Child Psychology and Psychiatry.

[CR54] Pears KC, Kim HK, Capaldi D, Kerr DCR, Fisher PA (2013). Father–child transmission of school adjustment: A prospective intergenerational study. Developmental Psychology.

[CR55] Phares V, Lopez E, Fields S, Kamboukos D, Duhig AM (2005). Are fathers involved in pediatric psychology research and treatment?. Journal of Pediatric Psychology.

[CR56] Pougnet E, Serbin LA, Stack DM, Schwartzman AE (2011). Fathers’ influence on children’s cognitive and behavioural functioning: A longitudinal study of Canadian families. Canadian Journal of Behavioural Science.

[CR57] Preston CC, Colman AM (2000). Optimal number of response categories in rating scales: Reliability, validity, discriminating power, and respondent preferences. Acta Psychologica.

[CR58] Rollins LS, Rollins LS (2020). Moving to father-inclusive services. Engaging and working with African American fathers: Strategies and lessons learned.

[CR59] Russell G, Barclay L, Edgecombe G, Donovan J, Habib G, Callaghan H, Pawson Q (1999). Fitting fathers into families: Men and the fatherhood role in contemporary Australia.

[CR60] Salinas A, Smith JC, Armstrong K (2011). Engaging fathers in behavioral parent training: Listening to fathers’ voices. Journal of Pediatric Nursing.

[CR61] Sarkies M, Long JC, Pomare C, Wu W, Clay-Williams R, Nguyen HM, Francis-Auton E, Westbrook J, Levesque J-F, Watson DE, Braithwaite J (2020). Avoiding unnecessary hospitalisation for patients with chronic conditions: A systematic review of implementation determinants for hospital avoidance programmes. Implementation Science.

[CR62] Scourfield J, Smail P, Butler D (2015). A systemic approach to improving the engagement of fathers in child safeguarding. Child Abuse Review.

[CR63] Scourfield J, Tolman R, Maxwell N, Holland S, Bullock A, Sloan L (2012). Results of a training course for social workers on engaging fathers in child protection. Children and Youth Services Review.

[CR64] Sharpe L, Jones E, Ashton-James CE, Nicholas MK, Refshauge K (2020). Necessary components of psychological treatment in pain management programs: A Delphi study. European Journal of Pain.

[CR65] Skulmoski G, Hartman F, Krahn J (2007). The Delphi method for graduate research. Journal of Information Technology Education: Research.

[CR66] Smith JD, Corace KM, MacDonald TK, Fabrigar LR, Saedi A, Chaplin A, MacFarlane S, Valickis D, Garber GE (2019). Application of the Theoretical Domains Framework to identify factors that influence hand hygiene compliance in long-term care. Journal of Hospital Infection.

[CR67] Stevens, B. J., Yamada, J., Promislow, S., Stinson, J., Harrison, D., Victor, J. C., & Members of the CIHR Team in Children’s Pain (2014). Implementation of multidimensional knowledge translation strategies to improve procedural pain in hospitalized children. Implementation Science : IS.

[CR68] Strike K, Chan A, Maly M, Solomon P (2019). Barriers to the implementation of point-of-care ultrasonography by physiotherapists in haemophilia treatment centres in Canada: A modified Delphi approach. The Journal of Haemophilia Practice.

[CR69] Taba P, Rosenthal M, Habicht J, Tarien H, Mathiesen M, Hill S, Bero L (2012). Barriers and facilitators to the implementation of clinical practice guidelines: A cross-sectional survey among physicians in Estonia. BMC Health Services Research.

[CR71] Tully LA, Collins DAJ, Piotrowska PJ, Mairet KS, Hawes DJ, Moul C, Lenroot RK, Frick PJ, Anderson VA, Kimonis ER, Dadds MR (2018). Examining practitioner competencies, organizational support and barriers to engaging fathers in parenting interventions. Child Psychiatry & Human Development.

[CR72] Tully LA, Piotrowska PJ, Collins DAJ, Mairet KS, Black N, Kimonis ER, Hawes DJ, Moul C, Lenroot RK, Frick PJ, Anderson V, Dadds MR (2017). Optimising child outcomes from parenting interventions: Fathers’ experiences, preferences and barriers to participation. BMC Public Health.

[CR73] Vamos CA, Thompson EL, Cantor A, Detman L, Bronson E, Phelps A, Louis JM, Gregg AR, Curran JS, Sappenfield W (2017). Contextual factors influencing the implementation of the obstetrics hemorrhage initiative in Florida. Journal of Perinatology.

[CR74] Vernon W (2009). The Delphi technique: A review. International Journal of Therapy and Rehabilitation.

[CR75] Vetere A (2004). Editorial: Are we continuing to neglect fathers?. Clinical Child Psychology and Psychiatry.

[CR76] Veugelers R, Gaakeer MI, Patka P, Huijsman R (2020). Improving design choices in Delphi studies in medicine: The case of an exemplary physician multi-round panel study with 100% response. BMC Medical Research Methodology.

[CR77] Vogel C, Zwolinsky S, Griffiths C, Hobbs M, Henderson E, Wilkins E (2019). A Delphi study to build consensus on the definition and use of big data in obesity research. International Journal of Obesity.

[CR78] Walsh VL, Fox LM, Brady M, King J, Worrell CM (2018). A Delphi consultation to assess indicators of readiness to provide quality health facility-based lymphoedema management services. PLoS Neglected Tropical Diseases.

[CR79] Waltz TJ, Powell BJ, Fernández ME, Abadie B, Damschroder LJ (2019). Choosing implementation strategies to address contextual barriers: Diversity in recommendations and future directions. Implementation Science.

[CR80] Wolins M, Lamb ME, Sagi A (1983). The gender dilemma in social welfare: Who cares for children. Fatherhood and family policy.

[CR81] Yamada, J., Squires, J. E., Estabrooks, C. A., Victor, C., Stevens, B., & CIHR Team in Children’s Pain (2017). The role of organizational context in moderating the effect of research use on pain outcomes in hospitalized children: A cross sectional study. BMC Health Services Research.

[CR82] Yap MBH, Pilkington PD, Ryan SM, Kelly CM, Jorm AF (2014). Parenting strategies for reducing the risk of adolescent depression and anxiety disorders: A Delphi consensus study. Journal of Affective Disorders.

