# Supplementary material for: Strategic Priorities for Implementation of Father-Inclusive Practice in Mental Health Services for Children and Families: A Delphi Expert Consensus Study
Source: Adm Policy Ment Health. 2022 Dec 19;50(4):538–51. doi: 10.1007/s10488-022-01222-1 (PMC10258177; doi:10.1007/s10488-022-01222-1)
Supplement: Supplementary file 1 — Supplementary file1 (DOCX 125 kb) [file 10488_2022_1222_MOESM1_ESM.docx]

**Supplementary Information**

**
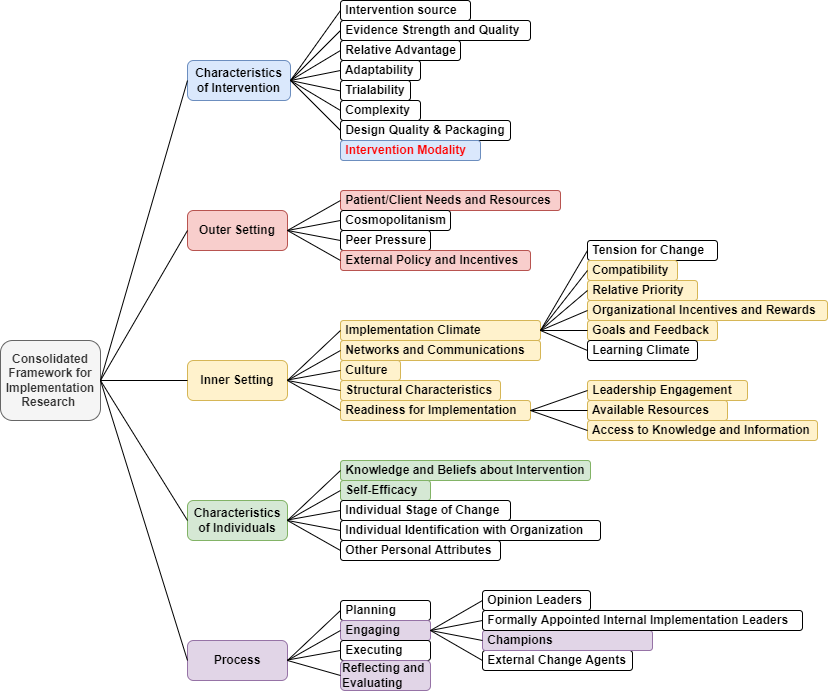
Figure S1. An overview of the CFIR framework used in the current study. Coloured boxes represent the constructs used to group the inductively derived barriers and facilitators to Father-Inclusive Practice.**

**Appendix S1.** Round 1 survey questions

1. **In your view, how much is father-inclusive practice considered by the services in organizational policies, recruitment, and support of staff and clients?** (We are looking for your expert opinion on what helps or hinders services from adequately considering fathers in their policies, recruitment and support)
2. **In your opinion, how much consideration is given by services to the timing, the place, and the format (e.g. online, face to face, group) of programmes and interventions to include fathers as well as mothers?** (Can you please elaborate on what you think the barriers and facilitators are for organizations to tailor their service delivery to fathers' needs?)
3. **What is your view on whether organizations provide sufficient resources to ensure reaching both fathers and mothers as part of service delivery?** (Please include your thoughts on what you think could stop services from reaching fathers? What do you think would help services to better reach fathers?)
4. **What is your opinion on whether services take sufficient steps to ensure that both mothers and fathers are informed about the importance of participation in family interventions, and benefits to children?** (Can you please elaborate on what you think the barriers and facilitators are for services to share this information with fathers as well as mothers?)
5. **To your knowledge, to what extent is data collection on parents who use services (e.g. monitoring of attendance, participation, and referrals) disaggregated by sex?** (Please elaborate on what you think stops organizations from recording the gender of the parents who use their services, and what could help with improving this practice?)
6. **In your opinion, how much emphasis do services put on training their practitioners to build skills to positively engage fathers?** (Can you please elaborate on what you think the barriers and facilitators are for organizations to **provide training for their practitioners to better engage fathers**
7. **What is your view on whether services provide sufficient resources to audit their father-inclusive practice and implement change toward greater father-inclusivity?** (Can you please elaborate on what you think the facilitators and barriers are for services to audit their father-inclusive practice and implement changes?)
8. In addition to your previous answers, do you have any other comments about what may help or hinder services from implementing organizational practices to better include fathers? Please share below.

**Appendix S2.** Round 2 survey questions

**Part 1: Please rate your agreement whether you think the following factors hinder child and family services from being more inclusive to fathers.** (Rated on a 7-point Likert Scale: Strongly Disagree (1) Disagree (2) Somewhat Disagree (3) Neither agree nor disagree (4) Somewhat Agree (5) Agree (6) Strongly Agree (7)).

1. Lack of training and education for the workforce on how to improve father inclusion

2. Organizations lacking sufficient financial resources to adequately fund father-inclusive practices

3. Lack of clear protocols that would help staff to adequately engage fathers

4. No rewards offered from service leadership for staff to be more inclusive of fathers in their practice

5. Services having limited awareness of fathers’ needs and how to address them

6. Limited staff availability due to inflexible working hours

7. Father-inclusive practices not being incentivized by the authorities and/or commissioners

8. Father-inclusive practices being viewed as creating additional work, resulting in excessive burden for staff

9. Lack of leadership’s commitment, engagement, and support for father-inclusive practice

10. Staff’s assumptions and stereotypes about fathers’ role, availability or interest in child and family interventions

11. Lack of confidence among child and family professionals in engaging dads

12. General organizational culture of resistance to change and reluctance to introduce new initiatives

13. No centralized guidance, i.e., father-inclusive practice being left at individual professional’s discretion

14. Lack of gender diversity in the staff teams, e.g., disproportionate percentage of female practitioners

15. Inadequate data recording systems, which don’t allow for storing and collating information that could enhance father inclusion

16. Reluctance to focus attention on fathers' needs because of the drive for child and family services to be gender-neutral

17. Father inclusive practices not being linked to service targets or key performance indicators

18. Lack of awareness of father-inclusive practice guidelines and recommendations

19. Father inclusion not being recognized as a strategic priority by services

20. Staff not having enough time for activities related to father-inclusive practice due to other competing demands

21. Staff’s assumptions that implementing father-inclusive practices will not lead to improved outcomes or increased father engagement

**Part 2: Please rate your agreement whether you think the following factors enable child and family services to be more inclusive to fathers.** (Rated on a 7-point Likert Scale: Strongly Disagree (1) Disagree (2) Somewhat Disagree (3) Neither agree nor disagree (4) Somewhat Agree (5) Agree (6) Strongly Agree (7)).

1. Services nominating champions committed to improving father inclusion

2. Services introducing targets and key performance indicators related to father-inclusive practice

3. Services providing more emphasis on remote treatment provision in efforts to better engage dads

4. Greater recognition of the importance of father-inclusive practice by the authorities and service commissioners

5. Services actively encouraging team discussion and reflection about implementing father-inclusive practice

6. Services introducing clear measures to monitor their provision of father-inclusive practice

7. Services providing opportunities for staff education and training on father-inclusive practice

Please use the space below if you would like to add further comments in relation to any of the questionnaire items.

**Appendix S3.** Round 3 survey questions

**Instructions included the following paragraph (including the table):**

“Below each question you will see feedback on how all participants responded, and you will be given a reminder of your own response from the previous round. This will take the format of a table - please see the example below: “

| Your previous rating was: | **Overall percentages from Round 2** | | | | | | |
| --- | --- | --- | --- | --- | --- | --- | --- |
|  | Strongly disagree | Disagree | Somewhat disagree | Neither | Somewhat agree | Agree | Strongly agree |
| **Agree** | **0%** | **0%** | **4%** | **2%** | **22%** | **35%** | **37%** |

**Part 1: Please rate your agreement whether you think the following factors hinder child and family services from being more inclusive to fathers.** (Rated on a 7-point Likert Scale: Strongly Disagree (1) Disagree (2) Somewhat Disagree (3) Neither agree nor disagree (4) Somewhat Agree (5) Agree (6) Strongly Agree (7))

1. Lack of training and education for the workforce on how to improve father inclusion

2. Organizations lacking sufficient financial resources to adequately fund father-inclusive practices

3. Lack of clear protocols that would help staff to adequately engage fathers

4. No rewards offered from service leadership for staff to be more inclusive of fathers in their practice

5. Services having limited awareness of fathers’ needs and how to address them

6. Limited staff availability due to inflexible working hours

7. Father-inclusive practices not being incentivized by the authorities and/or commissioners

8. Father-inclusive practices being viewed as creating additional work, resulting in excessive burden for staff

9. Lack of leadership’s commitment, engagement, and support for father-inclusive practice

10. Staff’s assumptions and stereotypes about fathers’ role, availability or interest in child and family interventions

11. Lack of confidence among child and family professionals in engaging dads

12. General organizational culture of resistance to change and reluctance to introduce new initiatives

13. No centralized guidance, i.e., father-inclusive practice being left at individual professional’s discretion

14. Lack of gender diversity in the staff teams, e.g., disproportionate percentage of female practitioners

15. Inadequate data recording systems, which don’t allow for storing and collating information that could enhance father inclusion

16. Reluctance to focus attention on fathers' needs because of the drive for child and family services to be gender-neutral

17. Father inclusive practices not being linked to service targets or key performance indicators

18. Lack of awareness of father-inclusive practice guidelines and recommendations

19. Father inclusion not being recognized as a strategic priority by services

20. Staff not having enough time for activities related to father-inclusive practice due to other competing demands

21. Staff’s assumptions that implementing father-inclusive practices will not lead to improved outcomes or increased father engagement

**Part 2: Please rate your agreement whether you think the following factors enable child and family services to be more inclusive to fathers.** (Rated on a 7-point Likert Scale: Strongly Disagree (1) Disagree (2) Somewhat Disagree (3) Neither agree nor disagree (4) Somewhat Agree (5) Agree (6) Strongly Agree (7))

1. Services nominating champions committed to improving father inclusion

2. Services introducing targets and key performance indicators related to father-inclusive practice

3. Services providing more emphasis on remote treatment provision in efforts to better engage dads

4. Greater recognition of the importance of father-inclusive practice by the authorities and service commissioners

5. Services actively encouraging team discussion and reflection about implementing father-inclusive practice

6. Services introducing clear measures to monitor their provision of father-inclusive practice

7. Services providing opportunities for staff education and training on father-inclusive practice

Please use the space below if you would like to add further comments in relation to any of the questionnaire items.

| **Table S1. A summary of proposed barriers and facilitators to the implementation of father-inclusive practice identified in Round 1 survey, grouped by themes and mapped onto the corresponding CFIR domains and constructs** | | | | |
| --- | --- | --- | --- | --- |
| **CFIR Domain** | **CFIR Construct** | **Descriptive inductive themes** | **Barrier** | **Facilitator** |
| Intervention characteristics | Intervention modality | Remote service provision | **-** | Services providing more emphasis on remote treatment provision in efforts to better engage dads |
| Outer setting | External policy and incentives | External incentives from authorities and commissioners  Drive for a gender-neutral approach to parent engagement | Father-inclusive practices not being encouraged or incentivized by the authorities and/or commissioners  Reluctance to focus attention on fathers' needs because of the drive for child and family services to be gender-neutral | Greater recognition of the importance of father-inclusive practice by the authorities and service commissioners  - |
|  | Patient/client needs and resources | Lack of awareness of fathers’ needs | Services having limited awareness of fathers’ needs and how to address them | - |
| Inner setting | Structural characteristics | High level of staff autonomy / lack of centralization. | No centralized guidance, i.e., father-inclusive practice being left at individual professionals’ discretion | - |
|  | Networks and communication | Promoting dialogue within services | - | Services actively encouraging team discussion and reflection about implementing father-inclusive practice |
|  | Organizational culture | Workplace culture resistant to change | General organizational culture of resistance to change and reluctance to introduce new initiatives | - |
|  | Implementation climate: compatibility | Burden associated with FIP implementation  Inadequate health data systems | Father-inclusive practices being viewed as creating additional work, resulting in excessive burden for staff  Inadequate data recording systems, which don’t allow for storing and collating information that could enhance father inclusion | -  - |
|  | Implementation climate: relative priority | Strategic prioritization of FIP | Father inclusion not being recognized as a strategic priority by services | - |
|  | Implementation climate: org. incentives and rewards | Internal incentives from leadership | No rewards offered from service leadership for staff to be more inclusive of fathers in their practice | - |
|  | Implementation climate: goals and feedback | Targets and key performance indicators | Father-inclusive practices not being linked to service targets or key performance indicators | Services introducing targets and key performance indicators related to father-inclusive practice |
|  | Readiness for implementation: leadership engagement | Leadership commitment to FIP | Lack of leadership’s commitment, engagement, and support for father-inclusive practice | - |
|  | Readiness for implementation: available resources | Time constraints  Availability of funding  Workforce diversity  Staff availability | Staff not having enough time for activities related to father-inclusive practice due to other competing demands  Organizations lacking sufficient financial resources to adequately fund father-inclusive practices  Lack of gender diversity in the staff teams, e.g., disproportionate percentage of female practitioners  Limited staff availability due to inflexible working hours | -  -  -  - |
|  | Readiness for implementation: access to information and knowledge | Education and training  Awareness of FIP guidelines and recommendations  Clear protocols for FIP | Lack of training and education for the workforce on how to improve father inclusion  Lack of awareness of father-inclusive practice guidelines and recommendations  Lack of clear protocols that would help staff to adequately engage fathers | Services providing opportunities for staff education and training on father-inclusive practice  -  - |
| Characteristics of individuals | Knowledge and beliefs about intervention | Staff’s assumptions and stereotypes  Lack of outcome expectancy | Staff’s assumptions and stereotypes about fathers’ role, availability or interest in child and family interventions  Staff’s assumptions that  implementing father-inclusive practices will not lead to improved outcomes or increased father engagement | -  - |
|  | Self-efficacy | Lack of confidence in engaging fathers | Lack of confidence among child and family professionals in engaging dads | - |
| Process | Engaging: champions | Establishing FIP champions in services | - | Services nominating champions committed to improving father inclusion |
|  | Reflecting and evaluating | Measures to monitor the provision of FIP | - | Services introducing clear measures to monitor their provision of father-inclusive practice (could also be – goals and feedback / reflecting and evaluating) |
